# A New Scoring System to Predict Lymph Node Metastasis and Prognosis After Surgery for Gastric Cancer

**DOI:** 10.3389/fonc.2022.809931

**Published:** 2022-02-07

**Authors:** Wen-Zhe Kang, Jian-Ping Xiong, Yang Li, Peng Jin, Yi-Bin Xie, Quan Xu, Yu-Xin Zhong, Yan-Tao Tian

**Affiliations:** Department of Pancreatic and Gastric Surgery, National Cancer Center/National Clinical Research Center for Cancer/Cancer Hospital, Chinese Academy of Medical Sciences and Peking Union Medical College, Beijing, China

**Keywords:** gastric cancer, lymph node metastasis, new scoring system, prognosis, surgery

## Abstract

**Background:**

Lymph node metastasis is one of the most important factors affecting the prognosis of gastric cancer patients. The purpose of this study is to develop a new scoring system to predict lymph node metastasis in gastric cancer using preoperative tests in various combinations of inflammatory factors and to assess the predictive prognosis value of the new scoring system for the postoperative gastric cancer patients.

**Method:**

This study includes 380 gastric cancer patients, 307 in the training set and 73 in the validation set. We obtain three inflammatory markers, CRA (C-reactive protein/albumin), SIRI (systemic inflammatory response index), and PLR (platelets/lymphocytes), by calculating and comparing the results of preoperative laboratory tests. By using these three indicators, a new scoring system is developed to predict lymph node metastases, assess patients’ prognoses, and compare clinicopathological characteristics in different patient subgroups. A nomogram is constructed to show and assess the predictive efficacy of every index for lymph node metastasis and survival.

**Results:**

In the new scoring system, higher scores are associated with more advanced pathological stage (p < 0.001), perineural invasion (p < 0.001), and vascular invasion (p = 0.001). Univariate and multivariable Cox regression analyses show that perineural invasion, vascular invasion, smoking history, and high scores on the new scoring system are significant risk factors for OS and RFS. High-scoring subgroups as an independent prognostic factor could predict overall survival (OS) and relapse-free survival (RFS). High scores on the new scoring system are significantly associated with the degree of lymph node metastasis (p < 0.001). CAR and PLR play very important roles in predicting lymph node metastasis in gastric cancer. CAR is a vital major marker in the prediction of patient survival.

**Conclusions:**

The new scoring system can effectively predict the patients’ lymph node metastasis with gastric cancer and can independently predict the prognosis of patients.

## Introduction

According to an epidemiological study, gastric cancer is one of the most common malignant tumors in China and also the 2nd highest overall incidence and mortality rate of malignant tumors, 67.91/100,000 and 49.80/100,000, respectively (1). Lymph node metastasis is a leading factor to the poor prognosis of patients with gastric cancer (2–5). Preoperative abdominal CT examination can diagnose lymph node metastasis in gastric cancer patients, but the accuracy still needs to be further improved. To improve the overall survival rate of gastric cancer patients, reliable biomarkers predicting lymph node metastasis can help confirm and make out high-risk patients, facilitate close follow-up of patients, and assist to develop appropriate treatment plans.

Systemic inflammation of tumor cell–host interactions is closely associated with tumor development and metastases among various malignancies (6–10). Based on previous studies, several pretreatment serum inflammatory markers including platelet–lymphocyte ratio (PLR), CRP albumin ratio (CAR), and systemic inflammatory response index (SIRI) are used to predict tumor progression and prognosis (11, 12). Serum albumin reflects the nutritional status of the patient. The progressive tumor invasion can lead to malnutrition, and the host’s response to the tumor can also lead to some changes in albumin levels (13). C-reactive protein is a highly specific marker of systemic inflammatory response and can reflect the stress state and inflammation level of the body (14). The CRP/Alb ratio is considered to reflect the nutritional status and inflammatory status of the patient. We speculate that combined analysis of the CRP/Alb ratio could predict lymph node metastasis and survival progress of patients after surgery. Systemic inflammation can affect tumor progression by inhibiting apoptosis, promoting angiogenesis, and damaging DNA (15). Platelets, neutrophils, monocytes, and lymphocytes are the four most representative blood components of inflammation in clinic. Siri and PLR obtained by calculating the ratio of these indexes can reflect the level of patients’ systemic inflammation; these two are also related to the prognosis of a variety of malignant tumors.

Currently, very few studies have examined the predictive role of preoperative inflammatory indicators in lymph node metastasis in gastric cancer. Therefore, this study retrospectively investigates the relationship between preoperative serum inflammatory markers and lymph node metastasis in gastric cancer and develops a new scoring system to assess the risk of lymph node metastasis in patients with gastric cancer as well as to predict survival outcomes after radical resection.

## Methods

### Participants

380 patients with gastric cancer who attended the Cancer Hospital of Chinese Academy of Medical Sciences for surgical treatment between September 2014 and March 2015 are included in this study; among them, 307 are in the training set and 73 are in the validation set. All patients were pathologically diagnosed as primary gastric cancer and underwent radical surgery before.

### Variables and Measurement

**Figure 1:**
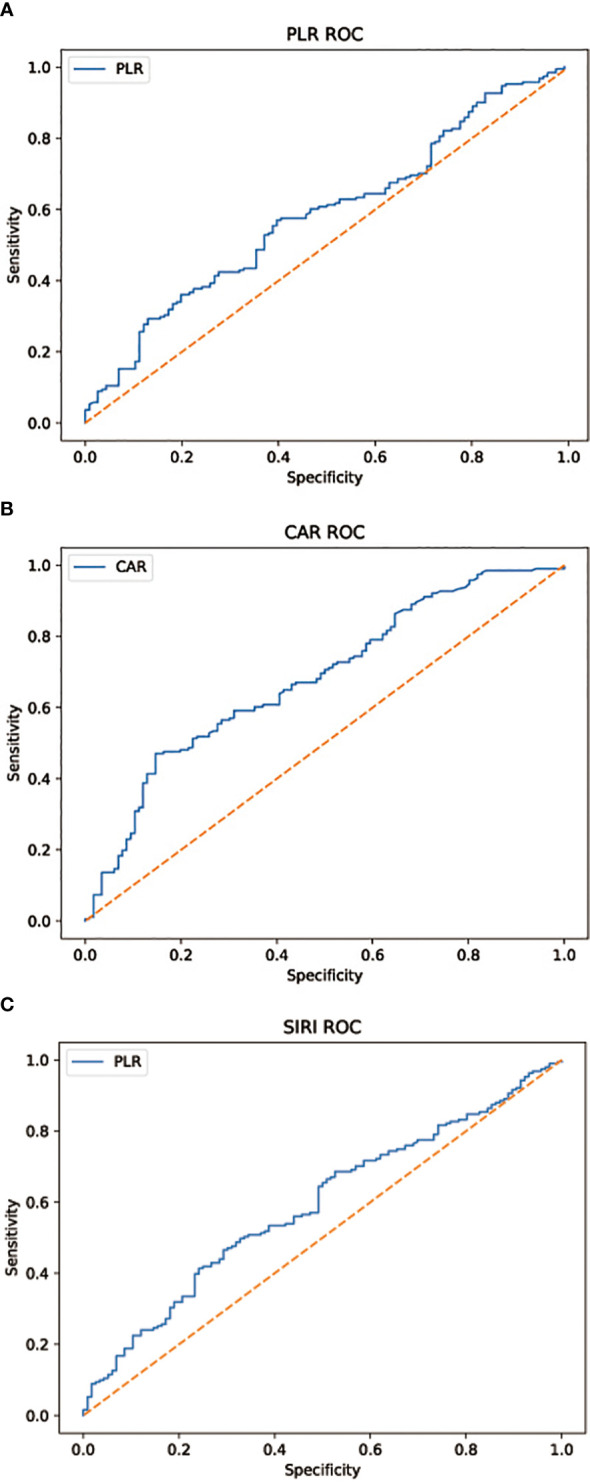
**(A)** AUC = 0.585, p = 0.012 **(B)** AUC = 0.680, p < 0.001 **(C)** AUC = 0.590, p = 0.008. Subject work characteristic (ROC) curve analysis to assess the predictive value of each combination of inflammatory factors for the discovery of lymph node metastasis in patients with gastric cancer in the cohort.

Blood samples from every patient are collected within 1 week prior to surgical treatment. Based on previous studies on tumor inflammatory markers, we select 3 typical inflammatory indicators (PLR, SIRI, CRA). We take advantage of these factors to develop a new scoring system for predicting lymph node metastasis in patients with gastric cancer and to assess the prognosis of patients. Youden’s index is adopted to establish the receiver operating characteristic curve (ROC) and thus to determine the optimal threshold values for the inflammatory indicators included in the study (**Figure 1**). The three indicators PLR, SIRI, and CRA are divided into two groups according to the optimal thresholds, with a score of “1” for the group above the optimal threshold and a score of “0” for the group below the optimal threshold. The 307 patients in the training set are assigned a score ranging from 0 to 3, with patients with scores of 0 and 1 divided into group “a” and patients with scores of 2 and 3 in group “b”. In the training set, we compare OS and RFS in group “a” versus group “b” and discuss the correlation of pathological features with the score results and prognosis. pN stage results were obtained from postoperative pathological reports. We specify 1 = N0; 2 = N1; 3 = N2; 4 = N3a; and 5 = N3b for the lymph node metastasis stage in postoperative pathological results. In the training set, we validate the relationship between the scoring results and the stage of lymph node metastases in the postoperative pathological findings. In the validation set, we verify the correlation between scoring results and the stage of lymph node metastases.

### Statistical Analysis

The ROC curve was established by calculating Youden’s index to determine the optimal threshold of inflammation indicators in this study. The chi-square test was used to analyze the relationship between the new scoring system and clinicopathological characteristics. Univariate and multivariable Cox regression analyses were performed to assess the impact of clinicopathological features on OS and RFS. Kaplan–Meier curves were used to describe survival in different score subgroups. p values were calculated by log-rank test. The relationship between the new scoring system and lymph node metastasis stage was analyzed by ordinal multicategorical logistic regression in the training set. In the validation set, we use linear regression to verify the correlation between the new scoring system and the lymph node metastasis stage.

Statistical analysis is performed using R software 4.0.5 (R Foundation for Statistical Computing, Vienna, Austria) and the SPSS 22.0 (SPSS Inc., Chicago, IL, USA). Test is bilateral, and a difference of p < 0.05 indicated statistical significance.

## Results

### Association of New Scoring System and Clinicopathological Characteristics

**Table 1** In the new scoring system, higher scores are associated with more advanced pathological staging (p < 0.001), perineural invasion (p < 0.001), and vascular invasion (p = 0.001).

**Table 1:** Association of new scoring system and clinicopathological characteristics.

Clinicopathological features	All cases	Group 0	Group 1	Group 2	Group 3	p value
Age						0.186
60≤	167	43	62	43	19	
60>	140	46	47	25	22	
Gender						0.359
Male	231	68	78	50	35	
Female	76	21	31	18	6	
PTNM stage						<0.001
I	102	45	40	13	4	
II	70	16	28	18	8	
III	135	28	41	37	29	
Tumor differentiation						0.857
G1	18	5	9	3	1	
G2	140	40	50	32	18	
G3	149	44	50	33	22	
Perineural invasion						<0.001
No	201	45	43	16	2	
Yes	106	44	66	52	39	
Vascular invasion						0.001
No	176	52	47	22	10	
Yes	131	37	62	46	31	
Smoking history						0.390
No	64	19	27	13	5	
Yes	243	70	82	55	36	
Family history of cancer						0.301
No	211	64	77	47	23	
Yes	96	25	32	21	18	

### Univariate and Multivariable Cox Regression Analyses of Clinicopathologic Variables in Relation to OS and RFS

Univariate Cox regression analysis shows that perineural invasion (HR: 5.721; 95% CI: 3.059–10.699; p < 0.001), vascular invasion (HR: 4.013; 95% CI: 2.461–6.543; p < 0.001), smoking history (HR: 8.446; 95% CI: 3.150–22.978; p < 0.001), and high scores on the new scoring system (HR: 1.627; 95% CI: 1.102–2.403; p = 0.014) are significant risk factors for OS. Multivariable Cox regression analysis is performed based on factors with p < 0.1 in the univariate Cox regression analysis. Perineural invasion (HR: 2.934; 95% CI: 1.368–6.295; p = 0.006), vascular invasion (HR: 2.584; 95% CI: 1.477–4.520; p = 0.001), smoking history (HR: 8.866; 95% CI: 3.250–24.189; p < 0.001), and high scores on the new scoring system (HR: 1.574; 95% CI: 1.064–2.328; p = 0.023) are identified to as significant independent risk factors for OS (**Table 2**).

**Table 2:** Univariate analysis and multivariable analysis of clinicopathologic variables in relation to OS.

Clinicopathological features	Case	Univariate analysis	p value	Multivariable analysis	p value
Age			0.570		
60≤	167				
60>	140				
Gender			0.201		
Male	231				
Female	76				
Tumor differentiation					
G1	18	Reference	0.392		
G2	140	3.201 (1.005–10.096)	0.049		
G3	149				
Perineural invasion			<0.001		0.006
No	201	Reference		Reference	
Yes	106	5.721 (3.059–10.699)		2.934 (1.368–6.295)	
Vascular invasion			<0.001		0.001
No	176	Reference		Reference	
Yes	131	4.013 (2.461–6.543)		2.584 (1.477–4.520)	
Smoking history			<0.001		<0.001
No	64	Reference		Reference	
Yes	243	8.446 (3.150–22.978)		8.866 (3.250–24.189)	
Family history of cancer			0.563		
No	211				
Yes	96				
New scoring system			0.014		0.023
Group a	198	Reference		Reference	
Group b	109	1.627 (1.102–2.403)		1.574 (1.064–2.328)	

Univariate Cox regression analysis shows that perineural invasion (HR: 5.805; 95% CI: 3.103–10.858; p < 0.001), vascular invasion (HR: 4.047; 95% CI: 2.481–6.600; p < 0.001), smoking history (HR: 8.285; 95% CI: 3.047–22.529; p < 0.001), and high scores on the new scoring system (HR: 1.636; 95% CI: 1.108–2.416; p = 0.013) are significant risk factors for RFS. Multivariable Cox regression analysis is performed based on factors with p < 0.1 in the univariate Cox regression analysis. Perineural invasion (HR: 3.259; 95% CI: 1.593–6.668; p = 0.001), vascular invasion (HR: 2.346; 95% CI: 1.341–4.104; p = 0.003), smoking history (HR: 8.262; 95% CI: 3.035–22.409; p < 0.001), and high scores on the new scoring system (HR: 1.588; 95% CI: 1.073–2.349; p = 0.021) are identified as significant independent risk factors for RFS (**Table 3**).

**Table 3:** Univariate analysis and multivariable analysis of clinicopathologic variables in relation to RFS.

Clinicopathological features	Case	Univariate analysis	p value	Multivariable analysis	p value
Age					
60≤	167		0.622		
60>	140				
Gender					
Male	231		0.079		
Female	76				
Tumor differentiation					
G1	18		0.334		
G2	140		0.115		
G3	149				
Perineural invasion			<0.001		0.001
No	201	Reference		Reference	
Yes	106	5.805 (3.103–10.858)		3.259 (1.593–6.668)	
Vascular invasion			<0.001		0.003
No	176	Reference		Reference	
Yes	131	4.047 (2.481–6.600)		2.346 (1.341–4.104)	
Smoking history			<0.001		<0.001
No	64	Reference		Reference	
Yes	243	8.285 (3.047–22.529)		8.262 (3.035–22.409)	
Family history of cancer					
No	211				
Yes	96				
New scoring system			0.013		0.021
Group a	198	Reference		Reference	
Group b	109	1.636 (1.108–2.416)		1.588 (1.073–2.349)	

### OS and RFS Examined on the Basis of New Scoring System

**Figure 2:**
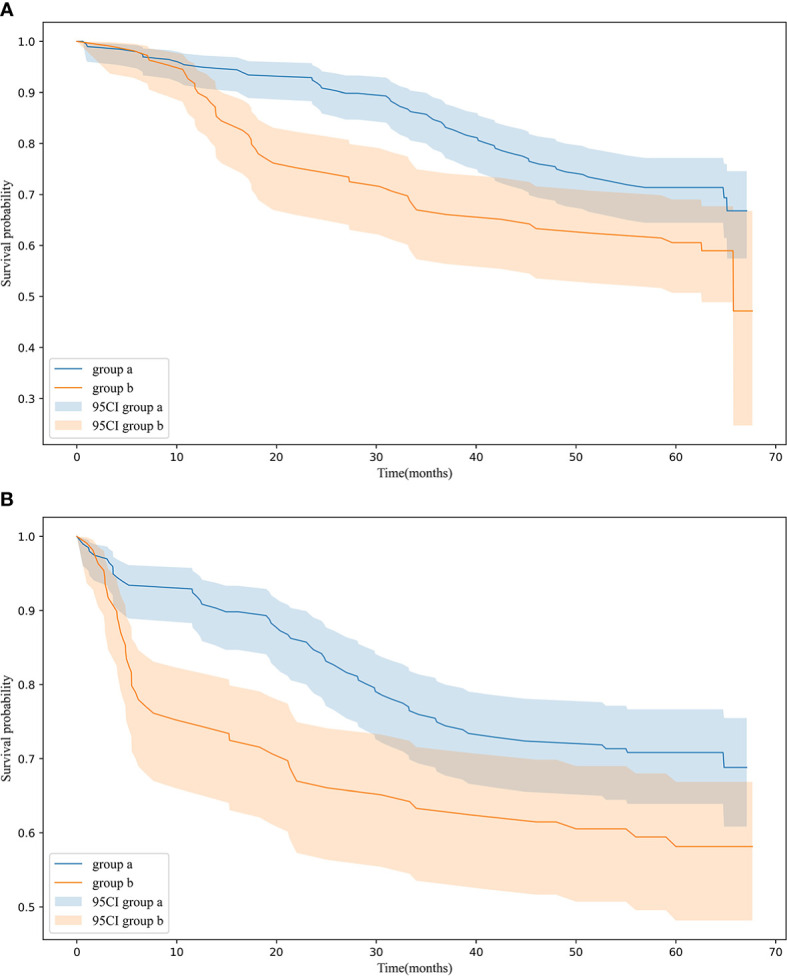
**(A)** OS in 198 patients (group a), 109 patients (group b) p = 0.013 **(B)** RFS in 198 patients (group a), 109 patients (group b) p = 0.012 OS and RFS for patients in group a and group b.

**Figure 2A**: The OS rates at 1, 3, and 5 years are 94.9%, 84.2%, and 71.3% for group “a” and were 89.9%, 66.1%, and 60.6% for group “b”. The average values of OS are 56.9 and 49.6 months for groups “a” and “b”. The Kaplan–Meier survival analysis indicates that a high score is related to poor OS for all included patients (p = 0.013).

**Figure 2B**: The average values of RFS are 53.9 and 45.7 months for groups “a” and “b”. The Kaplan–Meier survival analysis indicates that a high score is related to poor RFS for all included patients (p = 0.012).

### Predictive Value of Lymph Node Metastasis and Result of Nomograms

**Table 4**: Ordinal multicategorical logistic regression of validation set shows that high scores on the new scoring system are significantly associated with the degree of lymph node metastasis (**Table 4A**, p < 0.001). The risk of lymph node metastasis increased as the score increased (**Table 4B**).

**Table 4A:** Ordinal multicategorical logistic regression of validation set.

	Chi-square	df	p value
Model fitting information	31.063	3	<0.001

**Table 4B:** Parameter estimates.

		Estimate	p value
Threshold	p Lymph node stage 1	-1.573	<0.001
	p Lymph node stage 2	-0.657	0.022
	p Lymph node stage 3	0.105	0.712
	p Lymph node stage 4	1.454	<0.001
Location	New scoring system 0	-1.718	<0.001
	New scoring system 1	-1.136	0.001
	New scoring system 2	-0.562	0.011
	New scoring system 3	Reference	

**Table 5**: The linear regression results for the validation set shows that as the score of the patient scoring system increased, the degree of lymph node metastasis also tends to increase. There is a linear regression relationship between the degree of lymph node metastasis and patients’ scores in the scoring system (R = 0.589, R2 = 0.347, p < 0.001). The regression equation is Y = 0.438 + 1.070X.

**Table 5:** Linear regression of validation set.

R	R square	Adjusted R square	p value
0.589	0.347	0.338	<0.001

Y=0.438+1.070X.

**Figure 3:**
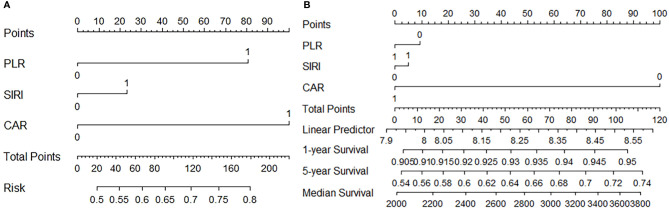
**(A)** Predictive value of PLR, SIRI, and CRA for lymph node metastasis **(B)** Predictive value of PLR, SIRI, and CRA for Survival.

**Figure 3A**: CAR and PLR play important roles in predicting lymph node metastasis in gastric cancer. **Figure 3B**: CAR is a major marker in the prediction of patient survival.

## Discussion

Many previous studies have demonstrated the potentials of various types of systemic inflammatory factors as markers for predicting prognosis of malignancies (16–20). Inflammation, malnutrition, and immune status are factors that affect tumor development and are closely related to the prognosis of gastric cancer patients (21). However, the long-term outcome of using combinations of systemic inflammatory factors detected in peripheral blood to predict lymph node metastasis and tumor prognosis in patients with gastric cancer has not been previously addressed in studies. In this study, a scoring system to predict lymph node metastasis is constructed using a combination of three preoperative systemic inflammatory factors and the prognostic impact of this score is evaluated, and several important findings are obtained in this study.

In the present study, we find that the scores in the scoring system consisting of preoperative serum inflammatory markers are significantly associated with the prognosis of patients with gastric cancer. CRP, a substance produced by the body in response to acute regulation of inflammatory cytokines in stress, is one of the most commonly used serum markers to assess the inflammatory status of patients and is identified as a prognostic indicator for patients with gastric cancer (22). Serum albumin is one of the most important indicators to assess the preoperative nutritional status of gastric cancer patients. Gastrointestinal obstruction and bleeding caused by gastric cancer can lead to malnutrition and increase the risk of postoperative complications in patients. Therefore, improving a patient’s preoperative nutritional status can bring a better survival outcome. Albumin is also a marker of systemic inflammation because the decrease in serum albumin levels is achieved through certain pro-inflammatory factors like cytokines (23). SIRI and PLR are two serum-based indicators of inflammation that have been shown in several studies as useful prognostic indicators for various types of cancer (24–28). Neutrophils and lymphocytes are associated with inflammation and immune activity, respectively (29). Platelets reflect the level of chronic inflammation in the body (27). The combination of these indicators has been shown effective in predicting survival outcomes in patients with gastric cancer (30–32). Lymphocytes can enhance immune surveillance of tumors and the inhibiting proliferation, invasion, and metastasis of tumor cells (33). Peripheral blood lymphocytes play a key role in the host cytotoxic immune response to tumors, and the systemic inflammatory response to host–tumor interactions may be a potential predictor of the development of distant metastasis in cancer patients (11). In this study, a scoring system is established by the systemic inflammatory response index of preoperative host–tumor interaction to predict the degree of lymph node metastasis in patients with gastric cancer, which can help to improve the preoperative assessment and guide the development of the next treatment plan. The new scoring system is validated in the validation set and confirmed as effective.

*Via* the nomogram, we noticed that CAR and PLR play very important roles in predicting lymph node metastasis in gastric cancer. The result suggests that preoperative serum albumin and platelet levels in patients with gastric cancer may be valuable predictors, and some studies have even discussed the association of these indicators with short-term postoperative complications in patients (34–37). In this study, we find that patients’ score results are significantly correlated with pathological stage, perineural invasion, and vascular invasion. It further suggests that the nutritional status of the patient and the level of systemic inflammation are closely related to the aggressive progression of the tumor. Univariate and multivariable Cox regression analyses of clinicopathological variables in relation to OS and RFS show that score outcomes significantly affected prognosis.

The new scoring system we developed incorporates nutritional and inflammatory indicators, allowing patients to receive nutritional support during preoperative interventions, thereby improving outcomes. The indicators needed in this study can be obtained during routine preoperative examinations without patients’ additional costs. Lymph node metastasis is a significant predictor of prognosis in patients with gastric cancer (38, 39). The strengths of this study are its ability to predict lymph node metastasis, its combination with imaging to assist in treatment planning, and its effectiveness in predicting patient survival outcomes. In clinical practice, this scoring system can be used as a supplement to TNM staging together with other scores and tests for the treatment of gastric cancer patients.

There are also some limitations in this study. Firstly, this is a single-center retrospective study, although strict inclusion criteria are established, but selection bias is inevitable. This study did not discuss gastric cancers originating from different sites separately. There are some differences between gastroesophageal junction carcinoma and gastric antrum carcinoma. Our study’s sampling size remains small, and patient characteristics, including tumor stages at enrollment, and patient ages, vary between these cohorts. This study is based on 3 screened representative inflammatory factors (SIRI, PLR, and CAR). Although we could successfully validate their clinical feasibility for the assessment of whether lymph node metastasis has been prognosed in patients with gastric cancer and treated with surgery, the accuracy, especially sensitivity, may still be insufficient as biomarkers for clinical screening. To overcome these limitations, larger prospective trials including ethnically diverse populations and consistent sampling protocols may be needed to further confirm the validity of these biomarkers and to assess their predictive potentials.

## Conclusion

Our study shows that preoperative inflammatory indicators are viable and promising predictive biomarkers. A new scoring system based on preoperative inflammatory indicators can effectively predict lymph node metastasis and postoperative predictive outcomes in gastric cancer patients. The scoring system we have developed can help physicians design more effective preoperative management and offer guidance for treatment strategies for patients with gastric cancer.

## Data Availability Statement

The original contributions presented in the study are included in the article/supplementary material. Further inquiries can be directed to the corresponding author.

## Ethics Statement

The studies involving human participants were reviewed and approved by the Institutional Review Board of Cancer Hospital, Chinese Academy of Medical Sciences, and Peking Union Medical College. The patients/participants provided written informed consent to participate in this study.

## Author Contributions

Y-TT designed the research. W-ZK, Y-XZ, J-PX, and PJ analyzed the data and wrote the paper. YL, Y-BX, and QX collected the patients’ clinical data. W-ZK and J-PX contributed equally to this work. All authors contributed to the article and approved the submitted version.

## Conflict of Interest

The authors declare that the research was conducted in the absence of any commercial or financial relationships that could be construed as a potential conflict of interest.

## Publisher’s Note

All claims expressed in this article are solely those of the authors and do not necessarily represent those of their affiliated organizations, or those of the publisher, the editors and the reviewers. Any product that may be evaluated in this article, or claim that may be made by its manufacturer, is not guaranteed or endorsed by the publisher.
